# Postbiotics in Functional Foods: Preparation-Based Characterization, Gut–Brain Axis Interactions, and Translational Perspectives

**DOI:** 10.3390/foods15142457

**Published:** 2026-07-10

**Authors:** Selin Elmas, Daniela Cîrțînă, Rodica Dîrnu, Ion Dorin Plută, Renata Maria Varut, Carmen Vladulescu, Adina Maria Kamal, Gabriela Pura, Romeo Popa, Denisa Daniela Sakizlian, Oana Diana Țîștea-Marcoci

**Affiliations:** 1 Independent Researcher, 10010 Balikesir, Türkiye; slnelmas92@gmail.com; 2 Department of Health and Motricity, Faculty of Medical and Behavioral Sciences, Constantin Brâncuși University of Târgu Jiu, 210185 Târgu Jiu, Romania; rodica.dirnu@e-ucb.ro (R.D.); dorin.pluta@e-ucb.ro (I.D.P.); denisa.mitroi@e-ucb.ro (D.D.S.); tistea-marcoci.diana@e-ucb.ro (O.D.Ț.-M.); 3 Research Methodology Department, Faculty of Pharmacy, University of Medicine and Pharmacy of Craiova, 200349 Craiova, Romania; 4 Department of Biology and Environmental Engineering, Faculty of Horticulture, University of Craiova, 200585 Craiova, Romania; carmen.vladulescu@edu.ucv.ro; 5 Department of Internal Medicine, University of Medicine and Pharmacy of Craiova, 200349 Craiova, Romania; adina.kamal@umfcv.ro; 6 Department of Medical Devices and Pharmaceutical Practice, Iuliu Hațieganu University of Medicine and Pharmacy, 400012 Cluj-Napoca, Romania; gabrielapura@gmail.com; 7 Department of Pharmacology, University of Medicine and Pharmacy of Craiova, 200349 Craiova, Romania; romeo.popa@umfcv.ro

**Keywords:** postbiotics, functional foods, gut–brain axis, food matrix, preparation characterization, immunomodulation, nutraceuticals, translational research

## Abstract

Postbiotics are defined as preparations of inanimate microorganisms and/or their components that confer a health benefit on the host. Although interest in postbiotics has increased substantially, their translational use in functional foods remains insufficiently characterized with respect to preparation identity, production methodology, food-matrix compatibility, mechanistic specificity, and regulatory positioning. This PRISMA-guided structured review aims to synthesize current evidence on postbiotics in functional food and nutraceutical contexts, with particular emphasis on preparation-based characterization, gut–brain axis-related mechanisms and clinical findings, food matrix applicability, and regulatory and health-claim considerations. Unlike broader postbiotic reviews that mainly address definitions, general health effects, or technological stability, this review integrates preparation identity, production process, gut–brain axis-related evidence, food matrix compatibility, and regulatory/health-claim translation within a single functional food framework. A structured literature search was conducted in Scopus and Web of Science Core Collection and was completed on 16 February 2026. The search strategy included three conceptual blocks: postbiotic and inactivation-based preparation terms, functional food/nutraceutical and food matrix terms, and gut–brain axis-related clinical and mechanistic terms. Cosmetic, topical, veterinary, animal feed, and aquaculture-focused publications were excluded. The export files contained 131 records from Scopus and 136 from the Web of Science Core Collection, yielding 267 records after applying document-type and language filters. After manually removing duplicates, 237 unique records were screened. Following title/abstract screening, 176 records were excluded as outside the scope of the review, and 61 publications were retained for full-text assessment and final thematic synthesis. The review was reported according to applicable PRISMA 2020 items. The evidence was organized into three thematic domains: gut–brain axis-related clinical findings, mechanistic evidence, and food matrix/product development applications. Heat-inactivated preparations, including *Lactobacillus gasseri* CP2305 and *Lactiplantibacillus plantarum* SNK12, have shown preliminary effects on stress-related symptoms, sleep quality, and selected neuroendocrine or inflammatory biomarkers in human studies. Mechanistic pathways include gut barrier integrity, immunomodulation, short-chain fatty acid signaling, tryptophan–kynurenine–serotonin metabolism, vagal communication, and regulation of the hypothalamic–pituitary–adrenal axis. Food matrix studies support the potential application of postbiotics in fermented dairy products, cereal-based systems, plant-based matrices, powders, concentrates, and bioactive packaging; however, matrix-dependent effects on bioavailability, sensory quality, and biological activity remain incompletely defined. Postbiotics provide a stable translational platform for functional-food development, but their scientific and commercial use requires clear characterization of the microbial source, production process, inactivation method, retained active fractions, dose metric, delivery matrix, and clinically meaningful endpoint. Future studies should avoid broad category-level claims and prioritize preparation- and matrix-defined human evidence with standardized safety reporting.

## 1. Introduction

The concept of functional food is defined not merely by a product’s nutrient composition but also by its ability to affect specific physiological functions through appropriate biomarkers and/or clinical endpoints. This European function-oriented approach is based on the premise that products should provide measurable health benefits beyond basic nutrition and that these benefits should be supported by mechanisms, biomarkers, and clinical outcomes [1,2]. This perspective is particularly relevant for microbiota-based functional foods because biological plausibility alone is insufficient unless the tested ingredient, preparation, matrix, dose, and outcome are clearly connected.

Microbiota-derived functional components have become increasingly important in functional food research due to their effects on gastrointestinal barrier integrity, immune-inflammatory responses, and metabolic signaling [3]. However, the efficacy of live probiotic applications varies considerably, depending on the preservation of cell viability, interactions with the product matrix, and sensitivity to processing and storage conditions. This variability contributes to heterogeneous clinical outcomes and reduces comparability across products [4,5]. These limitations have increased interest in microbial products that do not contain live microorganisms but may retain biological effects at the preparation level; such preparations offer potential advantages, including more predictable characterization, greater stability, and more consistent product standardization [6,7]. Nevertheless, postbiotics should not be viewed simply as “more stable probiotics.” Their interpretation requires preparation-level characterization, including the source microorganism, production process, inactivation or disruption method, preserved structural or soluble fractions, dose metric, and delivery matrix [8,9].

According to the current literature, postbiotics are defined as “preparations of inanimate microorganisms and/or their components that confer a health benefit on the host” [6]. This definition clearly indicates that purified single microbial metabolites should not be directly classified as postbiotics. Therefore, postbiotics should be considered holistic preparations defined not by a single metabolite or end product, but by their production method, inactivation technique, and behavior within the food matrix. Accordingly, terms such as postbiotic, paraprobiotic, cell-free supernatant, microbial metabolite, fermented-food component, and broader microbiome intervention should not be treated as interchangeable evidence categories. A study based on an isolated metabolite, a live probiotic, or a fermented food does not provide the same type of evidence as a study testing a defined inanimate microbial preparation. In recent years, the potential contributions of these preparations to the regulation of immune responses, support of barrier function, and modulation of metabolic signaling networks have been increasingly discussed [8,10,11].

One of the key areas in which postbiotics have gained attention is the gut–brain axis (GBA). The GBA refers to bidirectional communication between the gut and the central nervous system; the vagus nerve, hypothalamic–pituitary–adrenal (HPA) axis, immune responses, and tryptophan metabolism are among the major components of this network [3,12]. Evidence that fermented foods and microbiota-based interventions may influence mood, stress responses, neuroinflammation, and depression-related biological processes via this axis continues to accumulate [13,14]. Nevertheless, current human data remain limited and heterogeneous; differences in definitions of preparation, production methods, dose metrics, and outcome measures limit direct comparability. This limitation is especially important in the postbiotic field because GBA-related mechanisms derived from probiotic, fermented-food, animal, cellular, or metabolite studies should be regarded as mechanistic plausibility rather than direct postbiotic-specific clinical evidence unless the tested intervention is a defined postbiotic preparation.

Existing reviews have mainly addressed postbiotic definitions, general health effects, or selected mechanisms. Fewer syntheses have examined how preparation format, active fractions, food matrix behavior, gut–brain axis endpoints, human evidence, and regulatory substantiation interact in functional-food development. This review therefore evaluates postbiotics in functional foods by linking preparation identity, production approach, gut–brain axis evidence, matrix compatibility, and health-claim requirements. Its central premise is that functionality should be interpreted in the context of the defined preparation and final product, rather than by microbial taxonomy, metabolite presence, or product category alone.

## 2. Materials and Methods

**Figure 1:**
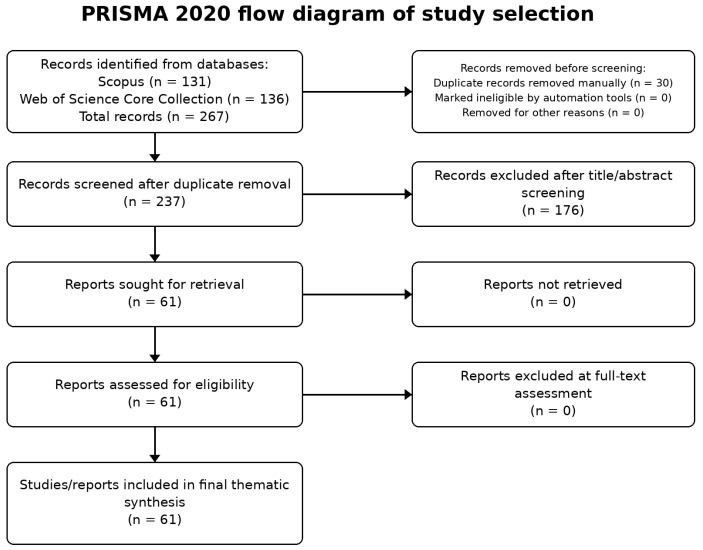
PRISMA 2020 flow diagram of study selection.

This study was designed as a structured narrative/scoping review examining the use of postbiotics in functional food and nutraceutical contexts, with a particular focus on effects related to the gut–brain axis (GBA). The literature search was conducted to identify, select, and thematically synthesize relevant evidence through a systematic search approach. Because the study did not aim to perform quantitative pooling, it did not follow a meta-analytic framework; instead, it focused on the conceptual, mechanistic, and application-oriented dimensions of the evidence. Reporting followed the PRISMA 2020 statement for systematic reviews without meta-analysis, where applicable [15]. The completed PRISMA 2020 checklist is provided as Supplementary Table S2, and the study-selection flow diagram is presented in Figure 1. No review protocol was prospectively registered; therefore, no registration number is available.

The literature search was conducted in Scopus and Web of Science Core Collection and was completed on 16 February 2026. English-language publications were considered, and document-type and language restrictions were applied before final export. In Web of Science, the search was performed in the TS (Topic) field using Advanced Search; this field covers titles, abstracts, author keywords, and Keywords Plus records. In Scopus, the search was performed in the TITLE-ABS-KEY field, which covers article titles, abstracts, and author keywords. The search strategy was structured around three conceptual blocks: (i) postbiotic and related preparation terms, (ii) functional food/nutraceutical and product matrix context, and (iii) gut–brain axis-related clinical and mechanistic terms. Cosmetic/topical applications and publications focused on veterinary feed or aquaculture were filtered using exclusion terms. The search strategy intentionally included broader preparation-related terms, such as paraprobiotic, cell-free supernatant, lysate, inactivated, heat-killed, non-viable, and microbial metabolite, to maximize retrieval sensitivity; however, these terms were not treated as conceptually equivalent during eligibility assessment. Final interpretation was restricted to postbiotics or postbiotic-relevant preparations consistent with the conceptual boundary of this review.

The main Web of Science search structure was as follows:

TS = (postbiotic* OR “post-biotic*” OR paraprobiotic* OR “para-probiotic*” OR metabiotic* OR “cell-free supernatant*” OR “cell free supernatant*” OR lysate* OR inactivated OR “heat-killed” OR “non-viable” OR “inanimate microbe*” OR “microbial metabolite*”).

AND TS = (“functional food*” OR nutraceutical* OR “functional ingredient*” OR “food matrix” OR “fermented food*” OR “dietary supplement*” OR yogurt OR kefir).

AND TS = (“gut-brain axis” OR “microbiota-gut-brain axis” OR “brain-gut axis” OR vagus OR “HPA axis” OR tryptophan OR kynurenine OR serotonin OR GABA OR BDNF OR neuroinflamm* OR “short-chain fatty acid*” OR butyrate OR propionate OR acetate).

NOT TS = (cosmetic* OR skincare OR topical OR dermatolog* OR poultry OR broiler* OR calf* OR swine OR pig* OR aquaculture OR fish* OR shrimp OR “animal feed” OR veterinary OR cat* OR dog*).

The corresponding Scopus search structure was adapted using the TITLE-ABS-KEY field as follows:

TITLE-ABS-KEY (postbiotic* OR “post-biotic*” OR paraprobiotic* OR “para-probiotic*” OR metabiotic* OR “cell-free supernatant*” OR “cell free supernatant*” OR lysate* OR inactivated OR “heat-killed” OR “non-viable” OR “inanimate microbe*” OR “microbial metabolite*”).

AND TITLE-ABS-KEY (“functional food*” OR nutraceutical* OR “functional ingredient*” OR “food matrix” OR “fermented food*” OR “dietary supplement*” OR yogurt OR kefir).

AND TITLE-ABS-KEY (“gut-brain axis” OR “microbiota-gut-brain axis” OR “brain-gut axis” OR vagus OR “HPA axis” OR tryptophan OR kynurenine OR serotonin OR GABA OR BDNF OR neuroinflamm* OR “short-chain fatty acid*” OR butyrate OR propionate OR acetate).

AND NOT TITLE-ABS-KEY (cosmetic* OR skincare OR topical OR dermatolog* OR poultry OR broiler* OR calf* OR swine OR pig* OR aquaculture OR fish* OR shrimp OR “animal feed” OR veterinary OR cat* OR dog*).

After the search results were exported, duplicate records were removed. Eligibility assessment was conducted in two stages: title/abstract screening and full-text review. Records retrieved from Scopus and Web of Science Core Collection were exported in BibTeX and tab-delimited plain-text formats, respectively. The export files contained 131 records from Scopus and 136 from the Web of Science Core Collection, yielding 267 records after applying document-type and language filters. Because the number of records was manageable, duplicate removal was performed manually rather than by automation software. Duplicate records were identified by comparing DOIs, titles, authors’ names, journals, and publication years. Thirty duplicate records were removed manually, leaving 237 unique records for title and abstract screening. No automation tools were used for eligibility exclusion.

The inclusion criteria were as follows: (1) the postbiotic or inactivation-based microbial preparation had to be clearly defined, for example through the use of inactivated cells, cell lysate, cell fragments, cell-free supernatant, or another defined non-viable microbial preparation; (2) the publication had to be conducted in a functional food/nutraceutical context and/or report at least one clinical, biochemical, technological, regulatory, or mechanistic outcome relevant to the gut–brain axis or functional food translation. The final synthesis included original human studies, experimental or mechanistic studies, product-development studies, and relevant review or regulatory publications when they contributed directly to the review’s conceptual or translational objectives. Publications focused exclusively on live probiotic applications; isolated microbial metabolites outside a defined postbiotic preparation context; cosmetic/topical applications; veterinary feed; aquaculture; or unrelated food technologies were excluded. Records were retained when they addressed postbiotics or postbiotic-relevant preparations, including heat-inactivated microorganisms, non-viable microbial cells, cell-free supernatants, microbial lysates, extracellular vesicles, or postbiotic-containing food matrices, and when they were relevant to functional foods, gut–brain axis mechanisms, food formulation, technological application, safety, translational development, or regulatory interpretation. Records were excluded when they focused exclusively on live probiotics, prebiotics, dietary fibers, polyphenols, fermented foods, or isolated microbial metabolites without a defined non-viable microbial preparation; when they were unrelated to functional food development or gut–brain/translational outcomes; or when the abstract indicated that the study did not provide mechanistic, technological, clinical, or regulatory information relevant to the review objectives.

Two reviewers independently screened titles and abstracts according to these predefined scope criteria. Disagreements were resolved through discussion and consensus; if agreement could not be reached, a third reviewer made the final decision. Based on this screening process, 176 records were excluded after title/abstract screening, and 61 publications were retained for full-text assessment. No additional full-text exclusions were recorded; therefore, 61 publications were included in the final thematic synthesis. The flow of record identification, duplicate removal, screening, eligibility assessment, and final inclusion is presented in Figure 1.

A standardized data extraction framework was used to record the preparation type, source microorganism, production or inactivation method, product matrix or application format, dose metric, duration of administration, comparator group, primary outcomes, GBA-related mechanistic markers, reported adverse events, and key methodological limitations. Data extraction was performed by two reviewers using this standardized extraction form, and the extracted items were checked for consistency. Any discrepancies were resolved through discussion and consensus. Findings were synthesized across three thematic axes: (i) GBA-related clinical findings, (ii) GBA-related mechanistic evidence, and (iii) food matrix/product development applications. Because this manuscript was designed as a structured narrative/scoping synthesis rather than a quantitative systematic review, no formal meta-analysis, pooled effect-size calculation, certainty-of-evidence grading, or assessment of reporting bias was performed. Risk of bias was not scored with a formal tool because the synthesis intentionally combined heterogeneous human, experimental, mechanistic, product-development, and regulatory evidence. Instead, the strength and relevance of the evidence was assessed descriptively according to study type, intervention definition, preparation characterization, product matrix, dose metric, comparator, population or model, endpoint type, adverse-event reporting, and main methodological limitations. Human randomized or controlled intervention studies were interpreted separately from animal, cellular, organoid, mechanistic, product-development, and regulatory evidence. This approach was used to avoid overinterpreting mechanistic biomarkers or preclinical findings as direct clinical evidence of postbiotic efficacy.

## 3. Conceptual and Technological Framework of Postbiotics

The concept of postbiotics has gained importance in the development of microbiota-based interventions in the functional food and nutraceutical fields, particularly given the limitations of viability-dependent approaches. In the literature, terms such as postbiotic, paraprobiotic, metabiotic, extracellular fraction, and microbial metabolite are sometimes used interchangeably, blurring conceptual boundaries. This terminology problem is not only semantic; it directly affects study selection, evidence interpretation, product classification, and health-claim substantiation. Therefore, in this review, terminology used for literature retrieval was separated from terminology used for conceptual classification [8]. The current definition shifts the focus from the mere presence of metabolites to the production conditions and functional properties of the preparation: biological effects should be evaluated not only according to the microbial source, but also according to the structural integrity of the preparation, its active fractions, and its behavior within the matrix [6].

A preparation-oriented approach is decisive for product standardization and translational applicability. The fragmented nature of the current scientific and commercial framework indicates the need for clearer standards regarding the definition, subcategories, quality criteria, and product-level characterization of postbiotics [16,17]. In this manuscript, postbiotic is used for a defined preparation of inanimate microorganisms and/or their components linked to a proposed or demonstrated host benefit; isolated metabolites are discussed only as postbiotic-associated or microbiota-derived mediators unless they are part of such a preparation [6,8,16]. Because culture conditions, substrate, inactivation method, retained fractions, storage, dose metric, and delivery matrix can change biological activity, these variables should be reported systematically.

Postbiotics used in functional food applications represent a heterogeneous group; inactivated whole cells, cell lysates, cell fragments, cell-free supernatants, and metabolite-rich fractions are among the main preparation formats [18,19]. Structural cellular components may interact more directly with host cells, whereas cell-free fractions may exert effects through soluble bioactives [19,20]. Short-chain fatty acids, organic acids, exopolysaccharides, bacteriocins, and bioactive peptides are among the main components of these fractions [21,22]. However, these compounds should not automatically be labeled as postbiotics when they are purified, chemically isolated, or discussed independently from a defined inanimate microbial preparation. In such cases, they are more accurately interpreted as microbial metabolites, postbiotic-associated components, or downstream mediators of microbiota activity. This diversity indicates that a single mechanism cannot explain postbiotic effects.

Preparations derived from the same microorganism should not be assumed to be biologically equivalent. A heat-inactivated cell preparation, a lysate, and a cell-free supernatant may differ in receptor interaction, epithelial or immune-cell contact, gastrointestinal stability, technological behavior, and dose standardization. Consequently, efficacy claims should not be extrapolated from one preparation format, inactivation protocol, or matrix to another unless directly supported by evidence [8,14,17,19,23]. Comprehensive overviews of postbiotics contribute to this framework, spanning discovery techniques through clinical applications [24]. Nevertheless, broad reviews should be interpreted with caution when they pool evidence from live probiotics, paraprobiotics, isolated metabolites, fermented foods, and defined postbiotic preparations without distinguishing among these categories.

One key variable determining the functional profile of postbiotics is the process used to prepare them [25]. Physical or chemical inactivation methods, such as heat treatment, pressure, ultrasound, and enzymatic disruption, not only eliminate proliferative capacity but may also affect cell wall integrity, surface proteins, extracellular polysaccharides, and soluble bioactive fractions to varying degrees [23]. Because these methods can preserve or disrupt microbial structures and soluble fractions in different ways, the term “inactivated” alone is insufficient for preparation-level interpretation. Two preparations obtained from the same strain under different processing conditions may display distinct chemical compositions and biological effect profiles [26]. Therefore, preparation characterization, process standardization, and matrix compatibility should be considered core components of product development in postbiotic research [27]. This point is particularly relevant for reviewer interpretation because efficacy claims cannot be extrapolated from one preparation format, inactivation protocol, or food matrix to another without direct supporting evidence.

The food matrix is an active determinant of postbiotic performance rather than a passive carrier. Matrix composition can affect active-fraction preservation, gastrointestinal release, bioavailability, sensory acceptability, and consumer adherence through pH, water activity, protein–polysaccharide interactions, lipid partitioning, storage stability, thermal tolerance, and digestion-driven release. For functional-food applications, efficacy should therefore be linked to the microbial origin, production process, and final product in which the preparation is delivered.

To avoid conceptual overlap between preparation types, related metabolites, and delivery matrices, the review separates postbiotic preparation formats from associated metabolites and food application contexts. Table 1A summarizes the main postbiotic preparation types and active fractions relevant to gut–brain axis mechanisms. In contrast, Table 1B summarizes related microbial metabolites and functional food delivery matrices, which should be interpreted as associated mechanisms or application contexts rather than as postbiotic categories in their own right.

## 4. Effects of Postbiotics Through the Gut–Brain Axis

The gut–brain axis is a bidirectional communication network between the gastrointestinal system and the central nervous system that operates through immune, endocrine, metabolic, and neural pathways. Gut barrier integrity, mucosal immune responses, microbiota-derived metabolites, tryptophan metabolism, the vagus nerve, and the HPA axis are among the principal mediators of this network [3,14]. A single biological pathway cannot explain the potential effects of postbiotics on this axis; rather, they arise from modulation of multiple signaling mechanisms that depend on the structural features of the preparation and its active fractions [32,41]. However, gut–brain axis evidence should be interpreted according to the level of evidence. Findings derived from broader microbiome, probiotic, fermented-food, animal, cellular, or organoid studies provide mechanistic plausibility. However, they should not automatically be interpreted as direct evidence of postbiotic efficacy in human functional-food interventions. The main mechanistic axes and related biomarkers are presented in Table 2.

### 4.1. Gut Barrier Integrity

Epithelial cells, the mucus layer, tight junction proteins, and the mucosal immune system play decisive roles in transmitting gut-derived signals to the systemic circulation and, indirectly, to the central nervous system. Disruption of barrier integrity may facilitate the translocation of microbial products and proinflammatory mediators into the circulation, thereby creating a physiological context associated with neuroinflammation and behavioral changes [33,42]. Available data suggest that some postbiotic preparations can influence barrier components such as ZO-1, occludin, and claudins and support mucosal defense; however, these effects vary by preparation type and experimental model [41,48]. Therefore, barrier-related endpoints should be interpreted as preparation- and model-dependent rather than as a general property of all postbiotics. In this context, a recent study using human-derived colon organoid tubules showed that a postbiotic derived from *Lactobacillaceae* improved TEER recovery and altered cytokine profiles, demonstrating the utility of advanced model systems for mechanistic barrier assessment [49]. Nevertheless, TEER recovery, tight-junction marker expression, and cytokine modulation remain mechanistic biomarkers unless they are linked to validated clinical outcomes in human intervention studies.

### 4.2. Immunomodulation and Neuroinflammatory Response

The gut–brain axis is not only a neural communication route but also a biological interface where peripheral and central immune responses intersect [3,33]. Microbiota components can be recognized at the receptor level by intestinal epithelial and immune cells, thereby influencing cytokine responses, inflammatory tone, and glial activation [41,43]. Suppression of low-grade systemic inflammation is considered an important intermediate mechanism underlying beneficial effects on the gut–brain axis [3,33]. Cell wall components, surface proteins, peptidoglycan derivatives, exopolysaccharides, and soluble bioactive molecules in extracellular fractions are particularly relevant in this context [41,43]. Because these components differ among intact inactivated cells, lysates, cell fragments, and cell-free supernatants, immunomodulatory effects should not be generalized at the species level alone. They should be interpreted in relation to the preparation format, production conditions, inactivation method, preserved active fractions, and host model used for evaluation. Butyrate has been reported to affect enteric neurons via G protein-coupled receptors such as FFAR3 (GPR41), thereby modulating vagal signaling and microglial activity; this supports a contribution of postbiotics to biological mechanisms underlying neuroinflammation and mood regulation [50,51]. However, butyrate-mediated signaling should be described as a microbiota-derived or postbiotic-associated mechanism unless the study directly evaluates butyrate within a defined postbiotic preparation.

### 4.3. Metabolic Mediators and Tryptophan Metabolism

Short-chain fatty acids, particularly acetate, propionate, and butyrate, are among the most frequently studied components of this axis because of their effects on epithelial integrity, immune responses, enteroendocrine signaling, blood–brain barrier function, and microglial activity; butyrate in particular has been associated with limiting neuroinflammation and regulating microglial responses [14,33,51]. In the context of postbiotic research, SCFAs should not be automatically classified as postbiotics in isolation. They may be interpreted as components of a defined postbiotic preparation, as postbiotic-associated metabolites, or as downstream microbiota-derived mediators depending on the experimental context. The tryptophan–kynurenine–serotonin axis is also a critical biochemical node; how tryptophan is directed among serotonin synthesis, the kynurenine pathway, and microbiota-related metabolic branches is an important determinant of the link between gut physiology and central nervous system function [44,45,52]. Accordingly, changes in tryptophan, kynurenine, serotonin, indole derivatives, or related ratios may provide mechanistic insight. However, they should not be presented as direct postbiotic effects unless they are linked to a defined preparation, dose metric, and relevant host outcome.

### 4.4. Neural and Endocrine Components

The vagus nerve can influence afferent activity via gut-derived metabolites and enteroendocrine signals; this pathway is particularly important for stress, anxiety, sleep, and autonomic regulation [3,45,53]. The HPA axis is especially relevant in the context of chronic stress: changes in intestinal permeability and inflammatory burden may affect HPA reactivity, with implications for cortisol responses, sleep regulation, emotional state, and cognitive function [46,47]. The potential of postbiotics in this domain is evaluated primarily using stress-related biomarkers and behavioral outcomes; however, current human data remain limited, and it is still too early to draw preparation-level, generalizable conclusions [32]. Therefore, claims related to stress, sleep, mood, cognition, neuroinflammation, or gut–brain axis modulation should be framed as preliminary and preparation-specific unless supported by controlled human intervention studies using clinically relevant endpoints. Outcomes such as cortisol, inflammatory markers, perceived stress scales, sleep indices, mood scores, and microbiota/metabolite profiles should be interpreted together rather than used as isolated evidence of efficacy.

## 5. Clinical Evidence: Critical Evaluation of Human Studies and Experimental Findings

Human data on the gut–brain axis-related effects of postbiotics remain limited and are largely based on specific strains and well-defined preparations. The outcomes most frequently assessed in the clinical literature are perceived stress, mood, sleep quality, selected gastrointestinal symptoms, and microbiota or metabolite indicators. There are very few postbiotic studies with adequate sample sizes conducted in defined clinical populations, such as major depression, anxiety disorders, neurodegenerative diseases, or cognitive impairment [14,32]. Therefore, the current human evidence should be interpreted as early preparation-specific evidence rather than as class-level clinical proof for all postbiotics. Meta-analyses of the broader bio-psychobiotic literature have evaluated the effects of biotics on depression, anxiety, and cognitive function and have reported promising but heterogeneous findings [54,55]. This meta-analytic context provides a useful benchmark for postbiotic-specific study designs. However, findings from probiotics, synbiotics, fermented foods, or broader psychobiotic interventions should not be directly extrapolated to postbiotics unless the tested product is a defined inanimate microbial preparation with an explicit dose metric, matrix description, and outcome linkage [9].

One of the most notable examples in human studies is the parapsychobiotic approach involving *Lactobacillus gasseri* CP2305. A randomized controlled trial reported that CP2305 use reduced stress-related symptoms and improved sleep quality [29]. Another randomized, double-blind, placebo-controlled study showed that the same preparation was associated with favorable changes in mood, sleep quality, and some gut microbiota indicators in young adults under chronic stress [56]. A systematic review and meta-analysis focused specifically on CP2305 further supports a sleep-related signal in adults, but also indicates that mechanistic confirmation and broader population-level validation are still needed [57]. A recent randomized controlled trial evaluating the effects of heat-inactivated *Lactiplantibacillus plantarum* SNK12 on sleep quality and stress-related neuroendocrine and inflammatory biomarkers demonstrated that postbiotic interventions can be examined using integrated designs that combine subjective endpoints with biological indicators [58]. These studies are valuable because they link defined preparations to measurable outcomes; nevertheless, they differ in strain, preparation process, dose, duration, population characteristics, and endpoint panels. These findings indicate that the effects are preparation-specific and cannot be generalized to the entire postbiotic class [32].

Studies on milk products fermented with *Lactobacillus paracasei* CBA L74 have reported changes in gut microbiota profiles and particularly in butyrate levels. However, the primary outcomes in these studies were not direct psychological or neurobehavioural endpoints. Therefore, these data carry important mechanistic value for the gut–brain axis but should not be interpreted as direct evidence of clinical neuropsychological efficacy [38]. They are better positioned as supportive translational evidence that a postbiotic-containing food matrix may modulate microbiota and metabolite profiles, rather than as proof of mood-, cognition-, or stress-related benefit. This distinction is important for health-claim interpretation because microbiota or metabolite modulation alone does not establish a clinically meaningful gut–brain outcome.

The main strengths of the current clinical evidence include findings suggesting that some postbiotic preparations are safe and tolerable under short-term use conditions, as well as multidimensional designs that go beyond subjective scales by jointly assessing sleep parameters, microbiota composition, and metabolite indicators [29,56,58]. Nevertheless, samples are mostly composed of healthy individuals or populations exposed to mild-to-moderate stress, and there is marked heterogeneity in product matrices, dose metrics, and biomarker panels. Many studies also remain limited by modest sample sizes, short intervention durations, variable comparator products, and inconsistent reporting of preparation characterization, inactivation procedures, viability confirmation, storage conditions, and matrix stability. Although studies evaluating the broader clinical potential of postbiotics in neuropsychiatric contexts remain limited, the literature examining neuropsychiatric therapeutic opportunities through the microbiota–gut–brain axis provides a translational framework for the field [59]. Overall, current human data allow only limited inferences [14,32]. At present, the strongest interpretation is that selected postbiotic preparations may influence stress- and sleep-related outcomes in specific populations. In contrast, evidence for major psychiatric, neurodegenerative, or cognitive disorders remains insufficient.

Preclinical studies provide a more detailed mechanistic picture. In animal and cell models, some postbiotic preparations have been reported to exert favorable effects on gut barrier function, inflammatory responses, oxidative stress, and behavioral outcomes. For example, some heat-inactivated *Lactobacillus* strains have been shown to reduce neuronal damage in oxidative stress models [60]. However, data from experimental models should be considered intermediate evidence supporting biological plausibility and mechanistic coherence rather than direct proof of clinical efficacy. Experimental findings are particularly useful for identifying active fractions, receptor-level interactions, barrier-related effects, cytokine profiles, oxidative stress pathways, and metabolite-mediated signaling. However, they should be translated cautiously because dose exposure, host physiology, microbiota context, and behavioral readouts in experimental models may not reproduce human functional-food use conditions.

**Figure 2:**
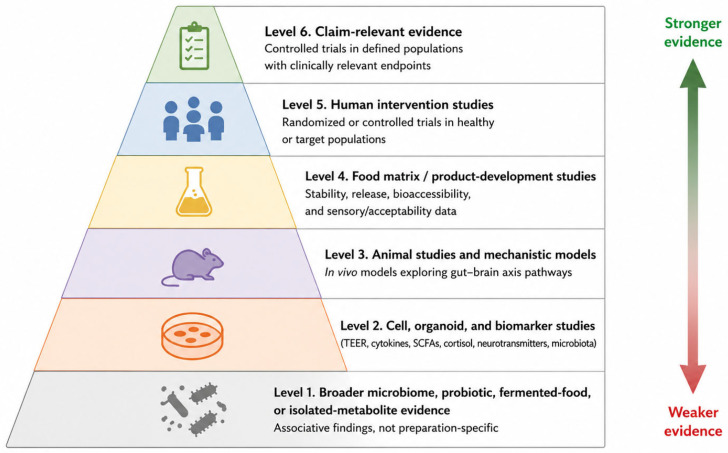
Evidence hierarchy for interpreting the postbiotic gut–brain axis.

Taken together, human and experimental data indicate that the most consistent gut–brain axis signals relate to stress response, sleep quality, barrier integrity, inflammatory tone, and microbiota/metabolite regulation. The current limitation is not only the number of studies, but also the limited differentiation of evidence by preparation, matrix, dose metric, and endpoint. Future trials should combine subjective psychological scales with inflammatory markers, tryptophan–kynurenine metabolism, short-chain fatty acid profiles, objective sleep assessment, and neurocognitive evaluation [25,32]. They should also predefine primary outcomes, register protocols, report adverse events, describe the preparation and matrix in detail, confirm non-viability when relevant, and avoid pooling distinct preparation formats without subgroup-level interpretation. Representative human, food-matrix/translational, and preclinical studies relevant to the postbiotic gut–brain axis are summarized in Table 3. The evidence hierarchy for interpreting these heterogeneous postbiotic gut–brain axis findings is shown in Figure 2.

## 6. Applications in Functional Food Matrices and Product Development Potential

One of the main reasons postbiotics have gained prominence in the functional food field is their ability to offer greater predictability and greater processing resilience than live probiotics. In live probiotics, preservation of biological efficacy depends on maintaining sufficient cell viability throughout storage; this is affected by numerous variables, including heat, oxygen, pH changes, and interactions with the product matrix. In contrast, appropriately designed postbiotics may be more resistant to food processing and storage conditions and may therefore provide important advantages in quality control, standardization, and shelf-life management [7,25,61]. However, greater processing resilience should not be interpreted as universal stability. Heat, acidity, oxygen exposure, water activity, packaging conditions, and storage duration may still alter soluble metabolites, peptides, extracellular polysaccharides, cell-wall structures, and other active fractions. Therefore, technological stability must be demonstrated for each preparation and matrix rather than assumed from the postbiotic label alone [62]. Production and application studies across various food matrices, including meat products, dairy systems, and cereal-based systems, indicate that postbiotics can exhibit consistent antimicrobial and technological performance across food categories [63]. These data support the technological potential of postbiotics, but product-level efficacy remains dependent on preparation format, concentration, processing conditions, storage stability, and the composition of the final food system.

The food matrix is not merely a passive carrier for postbiotics. The physicochemical structure of the matrix directly affects preservation of active fractions, gastrointestinal release profile, bioavailability, sensory acceptability, and consumer adherence. Therefore, the same preparation may produce different functional outcomes in different food formats. Matrix variables such as pH, buffering capacity, water activity, fat content, protein–polysaccharide interactions, phenolic compounds, salt concentration, thermal exposure, and packaging atmosphere may influence both technological performance and biological availability. From a functional food development perspective, the key issue is determining which matrix, dosage form, and consumption conditions enable sustainable delivery of biological activity. Product development should therefore integrate preparation characterization, matrix compatibility testing, simulated digestion or release assessment, sensory evaluation, shelf-life monitoring, and batch-to-batch quality control.

### 6.1. Dairy and Fermented Dairy Products

Dairy and fermented dairy products are among the most suitable matrices for postbiotic applications. Their strong historical association with microbial fermentation, consumer perception of them as healthy products, and their capacity to carry bioactive components are major advantages. Studies on products fermented with *Lactobacillus paracasei* CBA L74 have reported changes in gut microbiota composition and butyrate levels [38]. Yogurts fortified with postbiotic powders have also shown promising results in terms of sensory acceptance and shelf life [39]. For dairy matrices, the distinction between postbiotics generated in situ during fermentation and postbiotic preparations added as external ingredients should be clearly reported. This distinction affects dose calculation, process control, active-fraction preservation, and interpretation of the final product effect. In addition, protein structure, fat content, fermentation endpoint, acidity, cold-chain conditions, and storage duration should be evaluated, as they may influence texture, flavor, the release of bioactive fractions, and consumer adherence.

### 6.2. Cereal and Bread Systems

Bread, sourdough products, and cereal-based formats have the potential to reach a broader range of consumers. A study showing increased GABA content in sourdough bread enriched with *Lactiplantibacillus plantarum* H64 fermentate supports the feasibility of a “postbiotic bread” approach [36]. Studies examining the integration of attenuated probiotic cultures into food matrices have also reported promising findings for the development of cereal-based postbiotics [64]. The literature on starter culture development and innovation further strengthens the conceptual basis for this transformation in fermented foods [65]. In these systems, baking, water activity, and storage conditions are critical variables determining the activity of the preparation in the final product.

Cereal and bakery matrices require particular caution because thermal processing may inactivate microorganisms and modify heat-sensitive peptides, enzymes, volatile compounds, organic acids, and other soluble fractions. Therefore, the presence of a fermented substrate or an attenuated microbial preparation is not sufficient on its own; the retained active fractions and their stability after baking and storage should be documented. For bread and sourdough applications, regulatory interpretation may also depend on whether the microorganisms and fermentation process have a history of safe use in the relevant food category [64].

### 6.3. Plant-Based and Alternative Matrices

**Figure 3:**
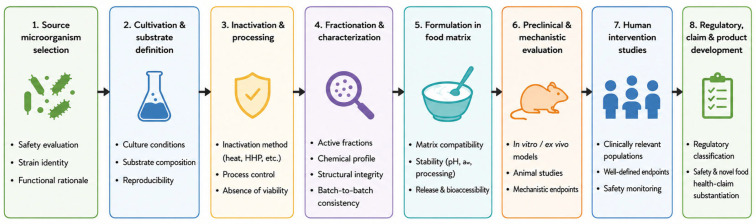
Preparation-to-product development framework for postbiotic functional foods.

Fermented plant-based systems may represent an important future platform for postbiotic applications because they align with clean-label and sustainability expectations. However, protein structure, buffering capacity, taste profile, and bioactive stability in these matrices differ from dairy-based systems; therefore, matrix-specific optimization is required for each preparation [40]. Plant proteins, fibers, phenolic compounds, organic acids, minerals, and antinutritional factors may interact with postbiotic fractions, thereby influencing solubility, release, sensory quality, and biological availability. For this reason, evidence obtained in dairy or supplement matrices should not be directly extrapolated to plant-based beverages, fermented vegetables, cereal drinks, or hybrid products without product-specific validation. Future studies should also consider consumer-relevant properties such as mouthfeel, off-flavor formation, color stability, clean-label positioning, allergen considerations, and compatibility with sustainable processing. Figure 3 summarizes the preparation-to-product development framework that connects postbiotic identity, matrix behavior, technological validation, human evidence, and claim substantiation.

When the existing literature is considered as a whole, successful use of postbiotics in functional foods requires that at least five elements be addressed together: (1) a clear definition of the preparation, (2) standardization of the production and inactivation protocol, (3) demonstration of matrix compatibility, (4) definition of a biologically meaningful dose metric, and (5) validation of sensory or technological performance. In response to current methodological limitations, these elements should be expanded into a product-development checklist that also includes confirmation of non-viability when relevant, description of active fractions, stability during processing and storage, release under gastrointestinal conditions, adverse-event monitoring, and evidence linking the final product to a relevant biological or clinical endpoint. Studies that fail to report these elements adequately make it difficult to determine which fraction or product condition is responsible for the health effect. Therefore, future translational studies should integrate preparation characterization, product engineering, and clinical validation on a single platform. Only this integrated approach can support credible functional-food development and prevent overgeneralized health claims based solely on microbial origin, fermentation status, or the presence of postbiotic-associated metabolites. The main carrier systems, advantages, and limitations of postbiotic applications in functional food matrices are summarized in Table 4.

## 7. Standardization, Regulation, and Health Perspective

The reliable evaluation of postbiotics in functional food and nutraceutical contexts requires that each preparation be clearly and reproducibly defined. Current expert consensus treats postbiotics not merely as “dead microbes” or “microbial metabolites” but as preparations associated with health benefits. Accordingly, scientific and regulatory assessment should be based on the specific preparation rather than on microbial origin alone. Essential information includes strain-level identity of the source microorganism, culture conditions, inactivation method, final composition, dose metric, and delivery matrix. For regulatory and health-claim interpretation, this preparation-level description should also include confirmation of non-viability when relevant; compositional specifications; active or marker fractions; manufacturing controls; stability data; intended use; target population; proposed daily intake; and the food or supplement matrix in which the preparation is delivered [70].

There is currently no independent, harmonized regulatory category for postbiotics in the European Union. Evaluation is conducted within the existing food legislation framework, taking into account the product’s nature and intended use. Regarding health claims, the main framework is the legislation governing nutrition and health claims on foods; this framework requires that claims not be misleading, that the relevant food or ingredient be sufficiently characterized, and that the claim be supported by scientific evidence capable of establishing a causal relationship with human health [71]. Therefore, regulatory acceptance of a postbiotic as a food ingredient, including through a novel food pathway, should not be confused with approval of a health claim. Safety, identity, and conditions of use are evaluated separately from the causal demonstration of a claimed physiological effect in humans.

If a postbiotic preparation has no meaningful history of consumption within the Union, a novel food assessment may also be required under Regulation (EU) 2015/2283. The approval of pasteurized *Akkermansia muciniphila* as a novel food in the EU represents an important precedent for regulatory acceptance of non-viable microbial products because it demonstrates that a pasteurized microbial preparation can be assessed as a defined novel food when identity, manufacturing process, viable-cell limits, composition, specifications, proposed uses, intake, and safety data are sufficiently documented [71,72]. The EU health claim roadmap proposed for prebiotics also provides useful guidance for postbiotics by illustrating how an integrated regulatory framework may be developed for functional biotic categories [73]. Nevertheless, postbiotic claims require evidence generated with the specific preparation rather than extrapolation from probiotics, fermented foods, prebiotics, or isolated microbial metabolites.

In the United States, postbiotics likewise lack an independent legal category. Whether the product is marketed as a conventional food or a dietary supplement determines the applicable regulatory pathway. This distinction is strategically important for postbiotic products positioned in areas such as the gut–brain axis, stress, and sleep. Structure/function claims must be notified within the required period, must not be misleading, and must not become claims for the treatment or prevention of disease [74]. Accordingly, wording such as “supports stress resilience,” “helps maintain normal sleep quality,” or “supports gut–brain axis balance” may require different substantiation and regulatory handling from wording that implies prevention, mitigation, or treatment of anxiety, depression, insomnia, neurodegeneration, or other diseases. For postbiotic products, claim strategy should therefore distinguish conventional food use, dietary supplement use, structure/function positioning, disease-claim boundaries, ingredient safety status, and the evidence needed to support the specific claimed effect.

The central challenge in developing health claims is that efficacy must be demonstrated not merely by microbial origin but also by the specific preparation. Regulatory systems rely less on general biological plausibility and more on well-defined product–outcome relationships. For this reason, evidence based only on taxonomic identity, in vitro biomarkers, animal models, microbiota shifts, or metabolite changes is generally insufficient for strong consumer-facing health claims unless it is supported by human intervention data using the final or clearly comparable preparation. Translational progress for postbiotics, therefore, appears to depend on strengthening preparation-based standardization, ensuring consistent nomenclature, and more tightly linking biomarkers to clinical outcomes in human intervention studies. Future regulatory dossiers should therefore integrate identity, manufacturing process, non-viability confirmation, active-fraction characterization, dose metric, matrix stability, safety, adverse-event monitoring, and clinically meaningful endpoints into a single product-specific evidence package.

## 8. Conclusions and Future Directions

Postbiotics have considerable translational potential in the functional food and nutraceutical fields. Current findings indicate that this potential is based not only on immunomodulation, support of barrier function, regulation of microbiota and metabolites, and gut–brain axis-related effects, but also on advantages related to product standardization, processing resilience, and shelf-life management. However, the available evidence is insufficient to treat all postbiotics as a single, homogeneous functional category. Scientific interpretation should therefore shift from broad taxonomic or ingredient-level claims toward evidence linking a defined inanimate microbial preparation, its active or marker fractions, its dose metric, its delivery matrix, and its intended physiological outcome.

The main priority in the field is not simply to expand conceptual interest but to generate stronger, clinically meaningful evidence. Human data on gut–brain axis outcomes remain largely limited to stress, sleep, and selected biomarkers. Future studies should use multilayered designs that combine clear definitions of preparation, standardized production and inactivation protocols, appropriate product matrices, biologically meaningful dose metrics, and well-defined clinical endpoints. Trials should also include adequate sample sizes, protocol registration, predefined primary outcomes, adverse-event monitoring, preparation characterization, confirmation of non-viability when relevant, storage and matrix-stability data, and endpoints that can be interpreted clinically rather than relying only on mechanistic biomarkers.

Meaningful progress will depend on demonstrating which preparation, produced by which method, delivered in which matrix, and directed toward which endpoint provides a measurable benefit. A scientifically defensible position in the functional food context will require preparation standardization, translational consistency, and well-designed human intervention studies. This approach is also essential for regulatory and health-claim translation because evidence from probiotics, fermented foods, isolated metabolites, animal models, or microbiota shifts cannot be directly extrapolated to postbiotic products without preparation-specific human evidence. Future research should integrate product engineering, mechanistic validation, clinical evaluation, safety assessment, and claim substantiation within a single development pathway.

## Figures and Tables

**Table 1:** (**A**) Postbiotic preparation types and active fractions relevant to gut–brain axis mechanisms. (**B**) Related microbial metabolites and functional food delivery matrices discussed as postbiotic-associated or application contexts.

**(A)**					
**Preparation Type**	**Main Active or Marker Fractions**	**Typical Use Format**	**Gut–Brain Axis Relevance**	**Interpretation Rule**	**Sources**
Heat-inactivated whole-cell/paraprobiotic preparations	Cell wall components, surface proteins, peptidoglycan derivatives, lipoteichoic acids, and structural fractions	Oral food, beverage, tablet, capsule, or powder formats	Stress, sleep, immunomodulation, and barrier integrity	It should be interpreted as postbiotic or postbiotic-relevant preparations only when the microorganism is non-viable/inanimate, the preparation is defined, and a host benefit is proposed or demonstrated.	[6,28,29]
Cell-free supernatants	Organic acids, soluble peptides, extracellular proteins, exopolysaccharides, and other soluble bioactive fractions	Oral or experimental format	Barrier function, immune response, and metabolic signaling	It should be interpreted at the preparation level. The term cell-free supernatant alone is insufficient unless the source organism, production conditions, processing, and bioactive fraction are described.	[6,7,19,30,31]
Cell lysates/cell-fragment preparations	Intracellular components, proteins, peptides, nucleic acid fragments, and structural microbial fragments	Oral or experimental format	Immunomodulation, barrier support, and receptor-level host interaction	Should not be treated as interchangeable with intact heat-inactivated cells or cell-free supernatants because preserved fractions and receptor interactions may differ.	[6,19,25]
Exopolysaccharide-rich preparations or fractions	Polysaccharide structures and associated microbial fractions	Oral or experimental format	Mucosal homeostasis and immune regulation	May represent a postbiotic-associated fraction when retained within or derived from a defined inanimate microbial preparation; purified EPS alone should be described more cautiously as a microbial-derived bioactive.	[21,22]
**(B)**					
**Conceptual Level**	**Component or Matrix**	**Main Feature**	**GBA/Functional Food Relevance**	**Interpretation Rule**	**Sources**
Related microbial metabolite	Short-chain fatty acids: acetate, propionate, and butyrate	Acetate, propionate, and butyrate	Barrier integrity, enteroendocrine signaling, neuroinflammation, and microglial activity	SCFAs are not postbiotics in isolation. They should be described as postbiotic-associated metabolites or downstream microbiota-derived mediators unless they are part of a defined inanimate microbial preparation.	[14,32,33]
Related microbial metabolite	Urolithin A	Gut microbial metabolite of ellagitannins	Mitochondrial function and neuroprotective potential	Should be treated as a microbiota-derived metabolite rather than a postbiotic preparation, unless explicitly incorporated into a defined inanimate microbial preparation.	[34,35]
Related bioactive/fermentation-derived component	GABA, organic acids, bioactive peptides, and bacteriocins	Neuroactive, antimicrobial, or fermentation-derived molecules	Neuroactive signaling, product functionality, and food preservation	These compounds may contribute to postbiotic-associated activity but should not be equated with postbiotics when purified or discussed independently.	[21,22,36,37]
Delivery matrix/functional food context	Fermented dairy products, including yogurt, kefir, and fermented milk	Mixed postbiotic fractions, organic acids, peptides, microbial cell components, and dairy matrix interactions	Microbiota profile, SCFA response, product development, and consumer adherence	This is a delivery or product matrix, not a postbiotic category. The preparation added to or generated within the matrix should be defined separately.	[38,39]
Delivery matrix/functional food context	Fermented cereal and bread systems	GABA, organic acids, and fermentation-derived bioactive fractions	Delivery of neuroactive components and product development	Matrix processing, especially baking and storage, may alter the active fractions retained; evidence should be interpreted at the product level.	[36,37]
Delivery matrix/functional food context	Plant-based and alternative fermented matrices	Phenolic transformation products, organic acids, microbial fractions, and plant matrix interactions	Sustainable product development and clean-label applications	This is a matrix/application context. Evidence from dairy or supplement formats should not be extrapolated without matrix-specific validation.	[23,40]

Note: The categories in Table 1A refer to preparation-level examples. They should be distinguished from isolated metabolites and from food matrices. Preparations derived from the same microorganism should not be assumed to be biologically equivalent, as the inactivation method, structural integrity, soluble fractions, dose metric, and final matrix may alter biological activity. The entries in Table 1B are not presented as postbiotic preparations. They are included because they are relevant to postbiotic-associated mechanisms, delivery formats, or functional food development. Isolated SCFAs, urolithin A, GABA, organic acids, peptides, bacteriocins, and fermented food matrices should not be classified as postbiotics unless they are part of a defined inanimate microbial preparation linked to host benefit.

**Table 2:** Main mechanistic axes, biomarkers, and related health outcomes associated with the gut–brain axis.

Mechanistic Axis	Main Biological Mediators	Postbiotic/Food Context	Assessable Biomarkers	Related Health Outcomes	Sources
Gut barrier integrity and mucosal homeostasis	Tight junction proteins, mucus layer, epithelial integrity	Heat-inactivated cell preparations, cell-free supernatants	ZO-1, occludin, claudins, MUC2, intestinal permeability indicators	Barrier preservation, reduced neuroinflammatory burden	[3,41,42]
Immunomodulation and neuroinflammatory response	Cytokine signaling, glial activation, peptidoglycan-mediated immune response	Cell wall components, lysates, EPS, and protein/peptide fractions	TNF-alpha, IL-1beta, IL-6, IL-10, CRP, oxidative stress markers	Suppression of low-grade inflammation	[3,33,43]
SCFA-mediated metabolic signaling	Acetate, propionate, butyrate; GPCR signaling; enteroendocrine response	Fermentation products, fermented dairy products	Fecal/serum SCFA profile, microbiota composition	Reduced neuroinflammation and metabolic balance	[11,32,33]
Tryptophan–kynurenine–serotonin axis	Tryptophan utilization, serotonin synthesis, kynurenine flux	Preparations associated with neuroactive metabolites	Tryptophan, kynurenine, serotonin levels; enzymatic pathways	Stress response, mood regulation	[44,45]
HPA-axis-related endocrine regulation	Cortisol response, stress hormones, and inflammatory–endocrine interaction	GBA-focused postbiotic preparations	Cortisol, stress scales, sleep parameters	Regulation of stress response, sleep, and emotional state	[32,46,47]

**Table 3:** Representative postbiotic studies in the context of the gut–brain axis: clinical and experimental evidence.

Evidence Level	Preparation/Intervention	Population or Model	Main Endpoints	Key Findings	Interpretation	Sources
Human RCT	*Lactobacillus gasseri* CP2305 parapsychobiotic preparation	Healthy students or adults exposed to academic or chronic stress	Stress-related symptoms, sleep quality, mood, gastrointestinal symptoms, and microbiota indicators	Improvements in selected stress-, sleep-, mood-, and microbiota-related outcomes were reported	Promising but preparation-specific evidence; not generalizable to all postbiotics or clinical psychiatric populations	[29,56]
Human RCT	Heat-killed *Lactobacillus helveticus* MCC1848	Healthy adults under transient stress conditions	Positive and negative affect, mood, sleep, fatigue, and quality-of-life indicators	Improvement was mainly reported in positive affect-related outcomes	Possible mood-related signal, but evidence remains limited by sample size, population type, and endpoint heterogeneity	[28]
Human RCT	Heat-killed *Lactiplantibacillus plantarum* SNK12	Healthy adults with sleep dissatisfaction or morning fatigue	Sleep quality, morning fatigue, cortisol, TNF-alpha, IL-6, and related biomarkers	Improvements in selected sleep-related outcomes and favorable changes in cortisol and TNF-alpha were reported	Useful integrated design combining subjective endpoints and biomarkers; confirmation in larger populations is needed	[58]
Food-matrix/translational evidence	Fermented milk product containing *Lactobacillus paracasei* CBA L74-derived components	Human or translational food context	Gut microbiota composition, butyrate levels, and selected metabolic or immune-related indicators	Changes in microbiota profile and butyrate levels were reported	Mechanistically relevant, but not direct evidence of clinical neuropsychological efficacy	[38]
Preclinical/mechanistic evidence	Heat-inactivated *Lactobacillus* strains or related postbiotic preparations	Cell, animal, oxidative stress, or neuronal injury models	Oxidative stress, neuronal damage, inflammatory markers, barrier-related or neuroprotective indicators	Favorable effects on neuronal injury, oxidative stress, or inflammatory pathways were reported in some models	Supports biological plausibility only; not direct proof of human clinical efficacy	[60]

Note: Human, food-matrix, and preclinical studies were not treated as equivalent levels of evidence. Detailed study-level information, including sample size, dose, duration, comparator, delivery form, adverse events, and main limitations, should be provided in Supplementary Table S1. Mechanistic biomarkers such as microbiota composition, SCFA profiles, cytokines, TEER, tight junction markers, cortisol, and inflammatory markers were interpreted as supportive evidence unless they were linked to clinically meaningful human endpoints.

**Table 4:** Applicability of postbiotics in functional food matrices: main carrier systems, advantages, and limitations.

Matrix Type	Representative Application	Technological Advantages	Main Limitations	GBA/Health Context	Sources
Dairy and fermented dairy products	Yogurt, kefir, fermented milk, and products enriched with postbiotic powders	High consumer acceptance, good carrier properties, and suitability for bioactive delivery	Sensory profile changes, heat treatment effects, fat/protein phase interactions	Strong translational basis for microbiota profile, SCFA response, and product development	[38,39]
Cereal and bread systems	Sourdough bread, cereal-based snacks, products containing fermentates	Wide consumption, low cost, and suitability for functional enrichment	Risk of bioactive fraction loss during baking and storage	Integration of neuroactive metabolites (GABA) into food matrices	[36,37]
Plant-based/alternative fermented matrices	Plant-based fermented beverages, alternative milk-like systems	Compatibility with clean-label and sustainability trends	Need for matrix-specific optimization; stability challenges	Potential platform for sustainable functional food development	[23,40]
Powder and concentrated formats	Postbiotic powders, dried fermented fractions	Does standardization, easy storage, and flexible formulation integration	Rehydration behavior and homogeneous distribution challenges	Controlled dose delivery and product development advantage	[25,39]
Comparative matrix use with live probiotics	Comparison of live probiotics and postbiotics in identical or similar product formats	No viability requirement, enabling more predictable quality control and process flexibility	The metabolic activity of live forms may provide additional benefits in certain contexts.	Useful for determining which product and target population are better suited to a postbiotic approach	[6,66,67,68,69]
Meat products and bioactive packaging	Meat, meat products, other food matrices, and bioactive packaging systems	Antimicrobial/antioxidant performance; potential shelf-life management through packaging	Matrix-specific stability, migration, sensory acceptance, and regulatory compliance requirements	Expanding area for product development aimed at food safety and quality preservation	[63]

## Data Availability

The original contributions presented in this study are included in the article/Supplementary Materials. Further inquiries can be directed to the corresponding author.

## Supplementary Materials

The following supporting information can be downloaded at: https://www.mdpi.com/article/10.3390/foods15142457/s1, Supplementary Table S1. Study-level details of representative postbiotic interventions and experimental evidence related to the gut–brain axis. Supplementary Table S2. Completed PRISMA 2020 checklist.
