# Supplementary material for: Postbiotics in Functional Foods: Preparation-Based Characterization, Gut–Brain Axis Interactions, and Translational Perspectives
Source: Foods. 2026 Jul 10;15(14):2457. doi: 10.3390/foods15142457 (PMC13408988; doi:10.3390/foods15142457)
Supplement: Supplementary file 1 [file foods-15-02457-s001.zip › foods-4376248-supplementary.pdf]

Supplementary Table S1. Study-level details of representative postbiotic interventions and experimental evidence related to the gut–brain axis.

Source	*Study design*	*Preparation / intervention*	*Population or model*	*Sample size*	*Matrix/delivery form*	*Dose or amount*	*Duration*	*Comparator*	*Primary endpoint / outcome domain*	*Adverse events / safety*	*Main limitations*
[24]	Randomized, double-blind, placebo-controlled human study	*Lactobacillus gasseri* CP2305 parapsychobiotic preparation	Healthy students under academic stress	n = 32 (21 M, 11 F)	Fermented milk-based beverage	1 × 10^10 counts/day	5 weeks	Lactic-acidified nonfermented milk beverage	Stress-related symptoms, sleep quality, gastrointestinal symptoms, and microbiota indicators	Not separately reported; GI symptoms monitored	Healthy student population; stress-exposure model; preparation-specific findings; limited generalizability
[47]	Randomized, double-blind, placebo-controlled human study	Heat-treated *Lactobacillus gasseri* CP2305	Healthy adults or students under chronic stress	n = 60 (CP2305 n = 31; placebo n = 29)	Tablet or supplement format	1 × 10^10 bacterial cells/day (two tablets)	24 weeks	Placebo tablets	Mood, sleep quality, stress-related indicators, and gut microbiota outcomes	No adverse events observed	Healthy population; stress-related model rather than clinical psychiatric disease; product-specific findings
[23]	Randomized, double-blind, placebo-controlled human study	Heat-killed *Lactobacillus helveticus* MCC1848	Healthy adults under transient stress conditions	n = 46 randomized; n = 43 completed; n = 39 PPS	Powder or supplement format	5 × 10^9 heat-killed cells/day	4 weeks	Placebo powder	Positive and negative affect, mood, sleep, fatigue, and quality-of-life outcomes	Mild/transient AEs; not test-food related	Small sample size; subjective endpoints; limited objective biomarker assessment; not a clinical population
[48]	Randomized, double-blind, placebo-controlled human study	Heat-killed *Lactiplantibacillus plantarum* SNK12	Healthy adults with sleep dissatisfaction or morning fatigue	SAF n = 55; efficacy set n = 50	Granulated preparation	≥1 × 10^11 cells/day (one 1-g packet)	4 weeks	Dextrin placebo granules	Sleep quality, morning fatigue, salivary cortisol, TNF-α, IL-6, and related biomarkers	No TEAEs; no clinically relevant safety changes	Short duration; healthy adult population; subjective sleep outcomes; biomarker findings require confirmation
[29]	Food-matrix / translational human or product evidence	Fermented milk product containing *Lactobacillus paracasei* CBA L74-derived components	Children 12–48 months; RCT substudy	n = 20 randomly selected children	Fermented milk product	7 g/day; product: 5.9 × 10^11 killed bacteria/100 g	3 months	Placebo/maltodextrin control	Gut microbiota composition, butyrate levels, and metabolic or immune-related indicators	Not reported in microbiota substudy	Primary outcomes were not direct psychological or neurobehavioral endpoints; matrix and preparation details require careful interpretation
[50]	Preclinical mechanistic study	Heat-inactivated *Lactobacillus* strains or related postbiotic preparations	Cell, animal, oxidative stress, or neuronal injury models	Not applicable to human sample size	Experimental preparation	Model-dependent	Model-dependent	Untreated or injury/stress control	Oxidative stress, neuronal damage, inflammatory markers, and neuroprotective indicators	Not applicable to clinical adverse-event assessment	Preclinical evidence; cannot be directly extrapolated to human functional-food efficacy

Note: Cells were completed from accessible source articles. Unreported or non-applicable information is stated explicitly.

Supplementary Table S2. Completed PRISMA 2020 checklist

Manuscript: Postbiotics in Functional Foods: Preparation-Based Characterization, Gut-Brain Axis Interactions, and Translational Perspectives

Note. The manuscript is a structured narrative/scoping synthesis with systematic search and selection procedures and no quantitative meta-analysis. Items specific to pooled effect estimates, quantitative synthesis, reporting-bias assessment, or certainty grading are marked not applicable where appropriate and justified in the Methods section.

PRISMA item	Topic	Location	Reported information / Notes
1	Title	Title page	The title identifies the topic and review focus. The abstract and Methods clarify the structured review design.
2	Structured abstract	Abstract: Background, Objective, Methods, Results, Conclusion	The abstract summarizes rationale, objectives, databases, date of search, screening counts, synthesis domains, main findings, and conclusions.
3	Rationale	Introduction	The Introduction explains why preparation-based characterization, matrix compatibility, gut-brain axis evidence, and translational positioning require synthesis.
4	Objectives	End of Introduction; Abstract Objective	The objective is to synthesize evidence on postbiotics in functional food/nutraceutical contexts with emphasis on preparation identity, GBA mechanisms, clinical findings, food matrices, and regulatory translation.
5	Eligibility criteria	Materials and Methods	Inclusion and exclusion criteria are specified for postbiotic or inactivation-based microbial preparations, functional food/nutraceutical context, GBA/translational relevance, and excluded live probiotic-only, isolated metabolite-only, cosmetic/topical, veterinary/feed, aquaculture, and unrelated food technology records.
6	Information sources	Materials and Methods	Scopus and Web of Science Core Collection were searched; the final search was completed on 16 February 2026.
7	Search strategy	Materials and Methods	Full Web of Science TS and Scopus TITLE-ABS-KEY search strings are reported, including postbiotic/preparation, functional food/matrix, GBA/mechanistic, and exclusion blocks.
8	Selection process	Materials and Methods; Figure 1	Two reviewers independently screened titles and abstracts; disagreements were resolved by consensus or third-reviewer adjudication. Full-text assessment and inclusion counts are reported.
9	Data collection process	Materials and Methods	A standardized extraction framework was used by two reviewers; extracted items were checked for consistency and discrepancies resolved by consensus.
10a	Data items: outcomes	Materials and Methods; Tables 2 and 3; Supplementary Table S1	Extracted outcomes included clinical endpoints, GBA-related mechanistic markers, product/matrix variables, adverse events, and methodological limitations.
10b	Data items: other variables	Materials and Methods; Supplementary Table S1	Extracted variables included preparation type, source microorganism, production/inactivation method, product matrix, dose metric, duration, comparator, population/model, and study limitations.
11	Study risk-of-bias assessment	Materials and Methods	No formal risk-of-bias tool was applied because the synthesis intentionally combined heterogeneous human, mechanistic, product-development, and regulatory evidence. Evidence relevance was assessed descriptively.
12	Effect measures	Not applicable	No meta-analysis or pooled effect-size estimation was performed.
13a	Synthesis: grouping eligibility for each synthesis	Materials and Methods	Findings were grouped into GBA-related clinical findings, mechanistic evidence, and food matrix/product-development applications.
13b	Synthesis: data preparation	Materials and Methods	No quantitative data transformation or statistical pooling was performed; evidence was synthesized descriptively by study type, preparation, matrix, and endpoint.
13c	Synthesis: tabulation/visual display	Tables 1A, 1B, 2, 3; Figures 1 and 2	Tables and figures summarize preparation categories, associated metabolites/matrices, mechanistic axes, evidence hierarchy, and representative studies.
13d	Synthesis: methods for results	Materials and Methods; Sections 4-7	Thematic narrative synthesis was used, separating human intervention evidence from mechanistic, preclinical, food-matrix, and regulatory evidence.
13e	Synthesis: heterogeneity	Materials and Methods; Sections 5 and 8	Heterogeneity was addressed descriptively according to preparation type, dose, matrix, population/model, endpoints, and study design; no quantitative heterogeneity statistic was applicable.
13f	Synthesis: sensitivity analyses	Not applicable	No quantitative synthesis or sensitivity analysis was performed.
14	Reporting-bias assessment	Not applicable	Formal publication/reporting-bias assessment was not applicable because no meta-analysis or effect-size pooling was performed.
15	Certainty assessment	Materials and Methods; Sections 5 and 8	No GRADE/certainty rating was applied. Evidence strength was discussed descriptively by study design, preparation definition, endpoint type, and translational relevance.
16a	Study selection results	Materials and Methods; Figure 1	The flow diagram reports records identified, duplicates removed, records screened, records excluded, reports retrieved/assessed, and final studies/reports included.
16b	Excluded studies/reports	Materials and Methods; Figure 1	No full-text exclusions were recorded after eligibility assessment; title/abstract exclusions are reported as outside review scope.

17	Study characteristics	Table 3; Supplementary Table S1	Representative human, food-matrix/translational, and preclinical/mechanistic evidence is summarized; study-level details are provided in Supplementary Table S1.
18	Risk of bias in studies	Not applicable / Materials and Methods	Formal study-level risk-of-bias results are not reported because no formal risk-of-bias tool was used; this limitation is stated and methodological limitations are discussed descriptively.
19	Results of individual studies	Section 5; Table 3; Supplementary Table S1	Key findings from representative human RCTs, food-matrix/translational studies, and mechanistic/preclinical evidence are summarized narratively and in tables.
20a	Results of syntheses: included studies per synthesis	Materials and Methods; Section 5; Figure 2	Evidence categories are explicitly separated into human, food-matrix/translational, preclinical/mechanistic, and broader mechanistic plausibility levels.
20b	Results of syntheses: quantitative results	Not applicable	No pooled quantitative synthesis was performed.
20c	Results of syntheses: heterogeneity results	Sections 5 and 8	Preparation-, dose-, matrix-, population-, endpoint-, and design-level heterogeneity is described narratively.
20d	Results of syntheses: sensitivity analyses	Not applicable	No sensitivity analyses were conducted because no pooled synthesis was performed.
21	Reporting biases	Not applicable	No formal reporting-bias assessment was performed; this is not applicable to the descriptive synthesis design.
22	Certainty of evidence	Sections 5 and 8	The manuscript provides a qualitative interpretation of evidence strength and limitations rather than a formal certainty rating.
23a	Discussion: general interpretation	Sections 5-8	Findings are interpreted in the context of preparation specificity, GBA mechanisms, food matrix applicability, safety, and regulatory translation.
23b	Discussion: limitations of evidence	Sections 5 and 8	The manuscript discusses limited human evidence, small samples, short durations, heterogeneous preparations, variable endpoints, matrix dependence, and insufficient clinical populations.
23c	Discussion: limitations of review process	Materials and Methods; Sections 5 and 8	The manuscript states that no meta-analysis or formal risk-of-bias scoring was performed and that evidence was synthesized descriptively.
23d	Discussion: implications	Sections 7 and 8	Implications for preparation standardization, matrix-specific validation, clinically meaningful endpoints, safety reporting, and health-claim substantiation are discussed.
24a	Registration information	Materials and Methods	No review protocol was prospectively registered; no registration number is available.
24b	Protocol access	Not applicable	No registered protocol is available.
24c	Protocol amendments	Not applicable	No registered protocol amendments are applicable.
25	Support	Funding statement	The manuscript states that no external funding was received.
26	Competing interests	Conflicts of Interest statement	The manuscript states that the authors declare no conflicts of interest.
27	Availability of data, code, and other materials	Data Availability Statement; Supplementary Materials	The manuscript states that contributions are included in the article and that the PRISMA checklist is provided as Supplementary Table S2.
